# Clinical Value of a 51-Neuroendocrine Tumor–Specific Gene Signature (NETest 2.0) for Management of Pancreatic and Small Bowel Neuroendocrine Tumors: A Prospective Study in a Consecutive Cohort of 82 Patients Considered for Surgery

**DOI:** 10.1097/AS9.0000000000000700

**Published:** 2026-08-10

**Authors:** Andrea Frilling, Ashley Clift, Duncan Spalding, Panagiotis Drymousis, Alexander von Roon, Robert Goldin, Jamshed Bomanji, Harpreet Wasa, Abdel B. Halim, Mark Kidd

**Affiliations:** From the * Department of Surgery and Cancer, Imperial College London, London, United Kingdom; † Cancer Research UK Oxford Centre, University of Oxford, Oxford, United Kingdom; ‡ Department of Surgery, Ealing Hospital, London North West University Healthcare NHS Trust, London, United Kingdom; § Department of Surgery, University College London Hospitals NHS Foundation Trust London, London, United Kingdom; ¶ Wren Laboratories, Branford, CT; ‖ Department of Nuclear Medicine, University College London Hospitals NHS Foundation Trust London, London, United Kingdom.

## Abstract

**Objectives:**

To prospectively evaluate the clinical utility of NETest2.0, a 51-neuroendocrine tumor (NET)–specific gene whole blood assay (NETest2.0) for monitoring surgical patients with gastroenteropancreatic NETs (GEPNET).

**Background:**

GEPNETs are challenging regarding postoperative surveillance and monitoring for recurrence or progression.

**Methods:**

Blood samples were collected at baseline and during follow-up, and NETest2.0 was measured (scored 0–100; cutoff normal: <50) in 82 consecutive patients with GEPNET. Scores were correlated with follow-up and disease status, including no evidence of disease, stable disease, recurrent disease, and progressive disease.

**Results:**

Pancreatic NET (n = 29): median age, 57 (26–81) years, M:F (17:12), G1: 19, G2: 8, G3: 2, stages I to III (n = 18), and stage IV (n = 11). Median follow-up, 122 (4–163) months; 7 (24%) died. NETest2.0 correlated with outcomes and was significantly higher in those who died (*P* = 0.0215). Small bowel NET (n = 53): median age, 66 (36–86) years, M:F (22:31), G1: 45, G2: 8, G3: 0, stages I to III (n = 25), and stage IV (n = 28). Median follow-up, 83 (12–157) months; thirteen (25%) died. NETest2.0 correlated with outcomes and was significantly higher in those who died (*P* = 0.0011). Scores ≥50 were significantly associated with recurrent disease (*P* = 0.0013). Scores ≥65 were associated with progression and poorer overall survival.

**Conclusions:**

NETest2.0 accurately identified disease status and correlated with clinical outcomes. Patients were stratified into molecular remission, stable disease, and high-risk progression states. Low scores (<50) identified surgical patients unlikely to recur; scores ≥65 identified individuals at increased risk for progression.

## INTRODUCTION

Gastroenteropancreatic neuroendocrine tumors (GEPNETs), recently reclassified under the overarching term neuroendocrine neoplasia, are clinically challenging. According to Surveillance, Epidemiology, and End Results Registries, the age-adjusted incidence of neuroendocrine tumor (NET) has increased 6.4-fold increase in the United States between1973 and 2012.^1^ The 20-year limited-duration prevalence increased from 0.006% in 1993 to 0.048% in 2012.^1^ Of the GEPNET originating from the pancreas or small bowel, about 50% to 80% are metastasized at initial diagnosis.

Surgical resection with an aim to radically eliminate the primary tumor, including locoregional metastatic disease, and, if applicable, complete resection of hepatic metastases, plays a central role in the treatment of GEPNET. A key clinical challenge is postoperative surveillance and monitoring for early recurrence (ER) or disease progression. Prediction of postsurgical course is challenging due to their biological heterogeneity and multiple clinicopathologic factors of potential prognostic relevance. For curative-intent resected grade (G) 1 or G2 pancreatic NETs (PanNETs), the recurrence rates are 12% to 17% with a median time to recurrence of 7 to 43 months.^2–4^ Overall survival (OS) is markedly lower among patients with PanNET with ER (within 18 months post-resection) compared with individuals with non-ER (42.5 vs 82.6 months; *P* < 0.01).^5^ Progression-free survival (PFS) was also significantly lower among ER patients (10.2 months) compared with non-ER patients (43.4 months). The data suggests that the timing of recurrence impacted both OS and PFS.

In small bowel NET (SBNET) resected with curative intent, reported recurrence rates amount to 31% to 42% with a median disease-free survival of 88 to 138 months.^6–8^ At highest risk of recurrence are patients with resected synchronous or metachronous liver metastases. While reports on their 5- and 10-year OSs are encouraging, recurrence rates are as high as 70% at 3 years and up to 94% at 5 years.^9,10^

Early identification of recurrence enables timely treatment prior to overt metastases and therapeutic protocols tailored to low tumor burden and fewer side effects. Although not clearly formulated in current guidelines, surgical patients at high risk for ER might benefit from neoadjuvant^11,12^ or adjuvant treatment concepts.^13^ In contrast, those identified with a low risk of recurrence could be recommended modified, less intensive follow-up protocols.

Current tools, for example, imaging and standard monoanalyte circulating tumor markers, remain suboptimal, especially for detecting ER or progression and persistent microscopic disease. Frequently recommended life-long imaging-based surveillance presents a significant economic burden and is associated with a risk of new cancers induced by exposure to ionizing radiation.^14^ A blood-based biomarker capable of dynamic molecular surveillance could enable earlier identification of recurrence or progression and rationalization of imaging frequency.

For the most used circulating tumor marker, chromogranin A (CgA), only a weak association exists between changes in plasma levels and the volume of tumor burden.^15^ Its value in assessing surgical resection effectiveness appears questionable.^16–18^ While specific secretory products and their metabolites are of value in functional NET, they are irrelevant for the majority.

Over the last 10 years, extensive research in applying liquid biopsy concepts, including circulating tumor cells, circulating tumor DNA (ctDNA), circulating free RNA, microRNA, extracellular vesicles, and other tumor-derived products as novel biomarkers in cancer, including NETs, has been performed.^19–25^

NETest2.0 is a quantitative reverse transcription polymerase chain reaction–based 51-circulating gene NET-associated mRNA assay combined with algorithmic output.^26,27^ The diagnostic accuracy and clinical value of the NETest in its original version (NETest 1.0) have been internally and externally validated in various studies and a meta-analysis.^28–31^ This multianalyte biomarker has demonstrated greater sensitivity and clinical utility than CgA for NET detection and management.^28,32^ Based on clinician and regulatory feedback, we have recently leveraged our well-established NETest protocol with contemporary supervised machine learning methods and expanded training and testing sets to improve the discrimination and calibration of the NETest algorithm (NETest2.0).^33^

In our previous work, we have demonstrated the efficacy of NETest (NETest 1.0) in the delineation of cytoreduction in patients with NET.^34,35^ The aim of this study was to assess the clinical value of NETest2.0 for monitoring of disease status, including postsurgical follow-up in a prospective cohort of patients with GEPNET. We hypothesized that normal NETest2.0 scores (<50) would have value in detecting surgically “cured” patients [no evidence of disease (NED)] and that increasing scores could be used to differentiate individuals with stable disease (SD) from progressive disease (PD). We also examined whether scores were prognostic for OS.

## METHODS

### Study Population

Consecutive patients with GEPNET seen between January 1 and December 31, 2011, at the Imperial College Health Care NHS Trust were invited to participate (Supplemental Figure S1, see https://links.lww.com/AOSO/A652). The diagnosis of an NET was established histologically on surgical specimens or on biopsy. All patients were discussed in multidisciplinary team meetings. Patients with PanNET (n = 29) or SBNET (n = 53) consented to participate in clinical research. Their data were collected in a prospectively maintained database. Ethics approval was obtained under 07/MRE09/54 (REC 22/WA/2836). Two of the authors (A.F. and A.C.) were responsible for the management of the patients and data collection since the beginning of the study. Standards for Reporting Diagnostic Accuracy Studies diagram, diagnostics, staging and grading, treatment, monitoring, and follow-up, as well as statistical approaches and methodology, are included in the Supplemental Digital Content.

### Blood Sample Collection for Research

Blood sampling at baseline and during follow-up was performed to enable longitudinal molecular surveillance and assessment of disease trajectory relative to imaging-defined status. Peripheral whole blood samples were collected, stored at −80 °C until shipped deidentified to Wren Laboratories, a clinically certified College of American Pathologists/Clinical Laboratory Improvement Amendments-testing facility. Deidentified results were correlated with clinical data. In patients who had surgery, preoperative blood samples were collected 24 hours before surgery or intraoperatively prior to induction of anesthesia. During follow-up, blood samples were collected as clinically appropriate. Samples for CgA were collected, but the high proportion of negative results (>70%) of samples precluded any formal evaluation of utility.

### Blood Sample Processing

Total RNA was isolated (RNeasy MiniKit: Qiagen, Valencia, CA; RNA quality ≥1.8 A_260:280_ ratio, mRNA production: 2.6–34 µg/sample).^36^ cDNA was synthesized (High-Capacity cDNA Reverse Transcriptase Kit: ThermoFisher Scientific; typical cDNA yield 2000–2500 ng/µL). Real-time PCR was performed on pre-spotted TaqMan PCR primer plates using 200-ng/µL cDNA/well.^37^ A positive control was included on each plate. Target transcript levels were quantified using ΔΔCT^36^ and data processed through the NETest2.0 algorithm.^33^ Results were expressed as a risk stratification score (0–100) for NET, which was converted into a binary readout (positive/negative). A cutoff score ≥50 was used to detect an NET.

## RESULTS

### PanNET (n = 29)

Demographics included M:F (17:12), median age, 57 (26–81) years, and grade: G1: 19, G2: 8, and G3: 2. Eighteen were stages I to III; 11 were stage IV. Median follow-up was 122 (4–163) months. Seven (24%) died (Table 1).

**Table 1.** Demographics and Clinicopathologic Characteristics: PanNET (n = 29)

Parameter	N (column % unless otherwise specified)
Total patients	29
Median age, y	57 (range, 26–81)
Sex	
Male	17 (58.6%)
Female	12 (41.4%)
Type of pancreatic tumor	
Nonfunctioning	21 (72.4%)
Nonfunctioning <2 cm in size	2 (6.9%)
MEN 1 associated	3 (10.3%)
Functioning	8 (27.6%)
Gastrinoma	3 (10.3%)
Insulinoma (benign)	2 (6.9%)
Insulinoma (malignant)	1 (3.4%)
Glucagonoma	1 (3.4%)
MEN 1–associated gastrinoma	1 (3.4%)
Tumor stage*	
Stages I and II (localized disease)	14 (48.3%)
Stage III (nodal disease only)	4 (13.8%)
Stage IV (distant metastases ± nodal disease)	11 (37.9%)
Liver metastases	11 (37.9%)
Synchronous	9 (31.0%)
Metachronous	2 (6.9%)
Tumor grade† (G)	
G1	19 (65.5%)
G2	8 (27.6%)
G3	2 (6.9%)
Surgical treatment	24 (82.8%)
Pancreato-duodenectomy	5 (17.2%)
Subtotal pancreatic resection‡	1 (3.4%)
Distal pancreatic resection	11 (37.9%)
Segmental pancreatic resection	5 (17.2%)
Enucleation	2 (6.9%)
Liver resection	4 (13.8%)
Nonsurgical treatment§	16 (55.2%)
Observation only‖	2 (6.9%)

* As per the AJCC staging system.

† As per the ENETS/WHO grading system.

‡ Parenchyma-sparing middle segment pancreatectomy, mainly in MEN 1 patients; in one patient, this was performed in combination with resection of a duodenal tumor.

§ As a component of multimodal treatment in addition to surgery or as a standalone nonsurgical treatment. At least once during the patients’ clinical course, including somatostatin analogues, peptide receptor radionuclide therapy, selective internal radiotherapy, transarterial chemoembolization, radiofrequency ablation, chemotherapy, and targeted medical therapy.

‖ Incidentally diagnosed nonfunctioning tumors <2 cm in size.

AJCC indicates American Joint Committee on Cancer; ENETS, European Neuroendocrine Tumor Society; MEN 1 indicates multiple neuroendocrine neoplasia 1; WHO, World Health Organization.

**FIGURE 1.**
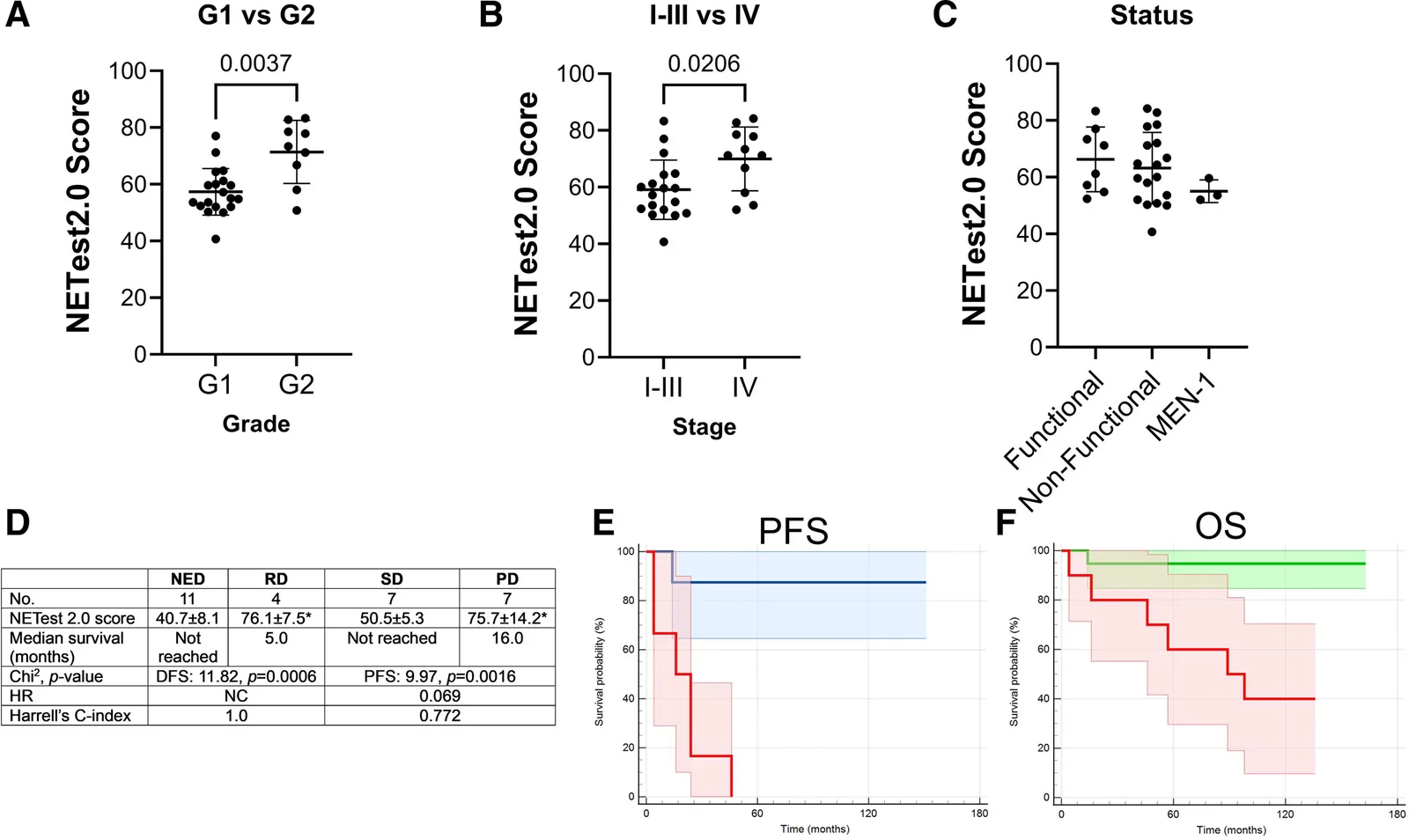
NETest2.0 scores in PanNET (n = 29). A, NETest2.0 scores differentiated by grade identify significant differences between G1 and G2. B, NETest2.0 scores were elevated in stage IV compared to stage I–III disease. C, No relationship was noted between tumor functionality and NETest2.0 scores. D, NETest2.0 scores and outcomes in PanNET. *P < 0.005. E, Kaplan-Meier survival scores identifying a cutoff of ≥65 significantly differentiated those with stable disease (blue) versus those who developed progression (red). F, The score cutoff of ≥65 was prognostic: survivors (green) versus those who perished (red). NC indicates not calculated.

Figure 1 includes NETest2.0 scores at first blood draw and the relationship to clinical parameters and outcomes. Scores were significantly higher in G2 tumors (Fig. 1A) and in individuals with stage IV disease (Fig. 1B). Tumor functionality appeared to have no relationship to scores (Fig. 1C). An evaluation of scores by type (NED vs residual disease) post-surgery identified scores were higher in those with residual disease (76.1 ± 7.5 vs 40.7 ± 8.1; *P* < 0.005; Fig. 1D). Using the normal cutoff identified 10/11 NED as <50 and 4/4 with residual disease as ≥50. Kaplan-Meier analysis on this set (n = 15) using this cutoff identified significant differences in outcome. None with NETest2.0 scores <50 developed image-detectable recurrence (up to 12-year follow-up), while those with positive scores developed detectable disease.

In those with detectable disease on imaging at the time of blood draw, similarly, higher scores were identified in those who developed progressive disease (75.5 ± 14.2) than those who remained stable (50.5 ± 5.3). We undertook AUROC analyses (Supplemental Fig. S2, see https://links.lww.com/AOSO/A652) and identified that the following NETest2.0 cutoffs (50, 55, 60, 65, 70, 75, and 80) may have value for predicting recurrence-free survival (RFS), PFS, and OS. For RFS, a cutoff of 50 was identified, while a cutoff of 65 was identified with balanced sensitivity/specificity metrics for both PFS and OS (Supplemental Fig. S2A–C and Table S1, see https://links.lww.com/AOSO/A652). Kaplan-Meier analysis on this set using a cutoff of 50 for RFS and 65 for PFS/OS identified significant differences in outcome. The median RFS was 8 (5–17) months in those with NETest2.0 scores ≥50 versus undefined (Supplemental Fig. S3, see https://links.lww.com/AOSO/A652).

None with scores <65 developed progression (up to 12-year follow-up), while those with scores ≥65 developed progression within a median of 16 months (Fig. 1E). The hazard ratio (HR) was 0.07 and χ^2^ = 9.97 (*P* < 0.002). We also examined OS in the PanNET group (n = 29). A cutoff of 65 accurately predicted outcomes. The median OS (mOS) was not reached (score < 65), while subjects with scores ≥65 perished within 89 months (Fig. 1F). The HR was 0.07 and χ2 = 10.42 (*P* < 0.001).

### Subanalyses: PanNET

Extended follow-up (≈12 years) was available for all patients. This has provided insights into the relationship between scores and individual patients and outcomes. Four patients had gastrinomas, one of whom was also a component of multiple neuroendocrine neoplasia 1. Scores were elevated (≥65) in 3, all 3 developed progressive disease, and 1 died. Three patients had insulinomas: 2 benign and 1 malignant. Benign disease was associated with low scores (<60) and stable disease through 12 years. The malignant insulinoma had highly elevated scores prior to surgery (>80) and remained with elevated scores post-R0 resection, consistent with a progressive disease journey. The patient with glucagonoma presenting with atypical symptoms and glucagon receptor mutations had low scores (<55) and has undergone a benign clinical course since complete resection (2012).^38^

The 3 patients with multiple neuroendocrine neoplasia 1 had benign courses and low scores, but the patient who developed progressive disease exhibited an elevated score (≥65) 2 years prior to the demonstration of progression. This was a 68-year-old woman who underwent a Whipple procedure. Her postsurgical score was 53.6 (NETest2.0-positive). She was considered NED. A year later, her score increased to 68 (+27%), and liver metastases were subsequently identified. A nonfunctional tumor example is a patient (female, 68 years) with a large G1 tumor initially treated with neoadjuvant chemotherapy, who underwent pancreatic resection with a liver resection and then peptide receptor radionuclide therapy. Within a year, the score increased to 92.3 (+59%) from the first postsurgical score of 58. A liver biopsy identified G2 metastasis. The patient subsequently died after failing courses of everolimus and sunitinib. NETest2.0 scores in patients with nonfunctioning PanNET <2 cm who were under surveillance were all low (<55), and none progressed during follow-up.

Notably, NETest2.0 scores <50 demonstrated a 100% negative predictive value for radiographic recurrence over the extended follow-up.

### Small Bowel NET (n = 53)

Demographics included M:F (22:31), median age, 66 (36–86) years, and grade: G1: 45, G2: 8, and G3: 0. Twenty-five were stages I to III, and 28 were stage IV. Median follow-up was 83 (12–157) months. Thirteen (25%) died from disease (Table 2).

**Table 2.** Demographics and Clinicopathologic Characteristics: SBNET (n = 53)

Parameter	N (column % unless otherwise specified)
Total patients	53
Median age, y	66 (range, 36–86)
Sex	
Male	22 (41.5%)
Female	31 (58.5%)
Primary tumor location	
Ileum	51 (96.2%)
Jejunum (all multifocal, with lesions also in the ileum)	2 (3.8%)
Tumor stage*	
Stages I and II (localized disease)	4 (7.5%)
Stage III (nodal disease only)	21 (39.6%)
Stage IV (distant metastases ± nodal disease)	28 (52.8%)
Liver metastases	28 (52.8%)
Synchronous	25 (47.2%)
Metachronous	3 (5.7%)
Tumor grade† (G)	
G1	45 (84.9%)
G2	8 (15.1%)
G3	0 (0.0%)
Surgical treatment	47 (88.7%)
Small bowel resection‡	36 (67.9%)
Right hemicolectomy‡	9 (17.0%)
Exploratory laparotomy and biopsy only	1 (1.9%)
Liver-free multivisceral transplantation	1 (1.9%)
Liver resection	8 (15.1%)
Nonsurgical treatment§	41 (77.4%)

* As per the AJCC staging system.

† As per the ENETS/WHO grading system.

‡ Including mesenteric lymphadenectomy.

§ As a component of multimodal treatment in addition to surgery or as a standalone nonsurgical treatment. At least once during the patients’ clinical course, including somatostatin analogues, peptide receptor radionuclide therapy, selective internal radiotherapy, transarterial chemoembolization, radiofrequency ablation, and mTOR inhibitors.

AJCC indicates American Joint Committee on Cancers; ENETS, European Neuroendocrine Tumor Society; mTOR, mechanistic target of rapamycin; WHO, World Health Organization.

**FIGURE 2.**
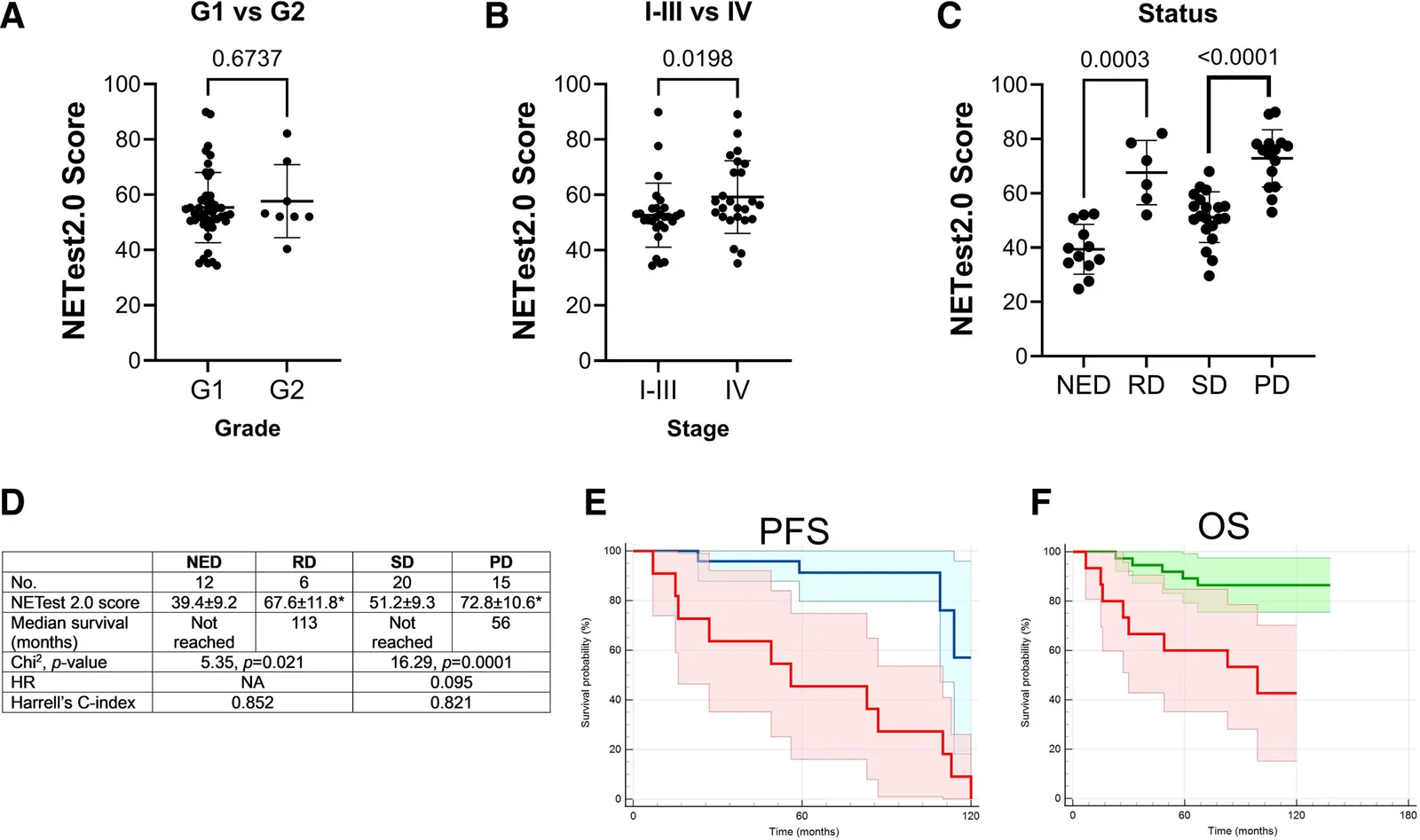
NETest2.0 scores in SBNET (n = 53). A, NETest2.0 scores were not different between G1 and G2. B, NETest2.0 scores were elevated in stage IV compared to stage I–III disease. C, Significant differences were noted between those with residual disease versus cures and between individuals with progressive versus stable disease. D, NETest2.0 scores and outcomes in SBNET. *P < 0.005. E, Kaplan-Meier survival scores identifying a cutoff of ≥65 significantly differentiated those with stable disease (blue) versus those who developed progression (red). F, The score cutoff of ≥65 was prognostic; survivors (green) versus those who perished (red). NC indicates not calculated.

Figure 2 includes NETest2.0 scores at the first blood draw and the relationship to different clinical parameters and outcomes. NETest2.0 scores were not significantly higher in G2 tumors (Fig. 2A) but were elevated (*P* < 0.02) in stage IV disease (Fig. 2B). Subjects who were NED after surgery had significantly lower scores than those with residual disease, while those with progressive image-detectable disease similarly had elevated scores compared to those considered stable (Fig. 2C). Scores were higher in residual disease (67.6 ± 11.8 vs 39.4 ± 9.2; *P* < 0.0005; Fig. 2D). Using the normal cutoff, 9/12 NEDs were <50, and 6/6 NEDs with residual disease were ≥50. Kaplan-Meier analysis on this set (n = 18) identified significant differences in outcome. None with scores <50 developed image-detectable recurrence (up to 12-year follow-up), while those with positive scores developed detectable disease with a medium RFS of 113 months.

In those with detectable disease at blood draw, similarly, higher scores were identified in those who developed progressive disease (72.8 ± 10.6) than those who remained stable (51.2 ± 9.3). Using the 65 cutoff (as used for PanNET) identified significant differences in outcome. The median PFS (mPFS) for those with NETest2.0 scores <65 was not reached, while those with scores ≥65 developed progression within a median of 56 months (Fig. 2E). The HR was 0.095 and χ^2^ = 16.29 (*P* = 0.0001). An evaluation of OS validated the cutoff of 65 (n = 53) as a prognostic. The mOS was not reached (NETest2.0 < 65), while subjects with scores ≥65 perished within a mOS of 99 months (Fig. 2F). The HR was 0.12 and χ^2^ = 10.38 (*P* < 0.002).

Exemplary examples include a 61-year-old female with a G2 tumor who underwent liver resection, then ileal and lymph node resections, and selective internal radiation therapy. Her score at treatment completion was 52. Within a year, it had increased to 78.3 (+51%) alongside disease progression on imaging; she started everolimus but died within 3 years. A second example is a 68-year-old male with a G2 tumor who underwent a right hemicolectomy. Despite being considered NED, his scores were positive post-surgery (52). His score increased to 72 (+38%); he was subsequently identified as having new lymph node metastases.

Of note, no patient with an NETest2.0 score <65 developed progression, supporting the assay’s value as a rule-out tool for disease activity.

### Multivariable Analyses

Multivariable regression analyses identified that NETest2.0 score was the strongest predictor of RFS (*P* < 0.0001), and NETest2.0 score was independently associated with increased hazard [HR, 1.07 per unit increase (95% CI, 1.026–1.12); *P* = 0.0018; Supplemental Tables S2 and S3, see https://links.lww.com/AOSO/A652].

For PFS NETest2.0 scores were the only independent predictor (β = 0.0193; SE = 0.0027; *P* < 0.0001) in the entire cohort (Supplemental Table S4, see https://links.lww.com/AOSO/A652). This was reflected in the results from Cox regression analysis: NETest2.0 score was independently associated with increased hazard [HR, 1.08 per unit increase (95% CI, 1.047–1.114); *P* < 0.0001; Supplemental Table S5, see https://links.lww.com/AOSO/A652]. No other clinical variables, including tumor grade, primary site, lymph node involvement, or liver metastases, were significantly associated with PFS.

For OS, both NETest2.0 score and liver metastases were identified as independent predictors (Supplemental Table S6, see https://links.lww.com/AOSO/A652). NETest2.0 score demonstrated a statistically significant positive association with the outcome (β = 0.009; *P* = 0.005), and liver metastases were significantly associated with OS (β = 0.279; *P* = 0.0135). For OS, in the multivariable Cox proportional hazards model, NETest2.0 score and liver metastases were identified as independent predictors of OS (Supplemental Table S7, see https://links.lww.com/AOSO/A652). NETest2.0 score was significantly associated with an increased hazard of death [HR, 1.056 (95% CI, 1.016–1.098); *P* = 0.006], indicating that each unit increase in NETest2.0 score corresponds to an ≈5.6% increase in mortality risk. Patients with liver metastases had more than a fourfold increased risk of death compared to those without. No other factor was associated with the outcome.

### Data Validation and Summation

As a mechanism to validate the score clinically, we compared area under the curves (AUCs) for differentiating NED/recurrent disease (RD) and PD/SD between the PanNET cohort (n = 29) and the SBNET cohort (n = 53) and also OS. For molecular surveillance classification (NED vs RD), AUCs were 0.955 ± 0.046 versus 0.875 ± 0.065, respectively. This was not significantly different (z-statistic: 1.0083; *P* = 0.313). For SD/PD, AUCs were 0.929 ± 0.0714 versus 0.867 ± 0.059, also not significantly different (z-statistic: 0.66833; *P* = 0.504). Similarly, there were no differences in OS (AUC, 0.838 ± 0.0829 vs 0.718 ± 0.0768; z-statistic: 1.0719; *P* = 0.288).

Supplemental Figure 4, see https://links.lww.com/AOSO/A652, summarizes the data for the combined pancreatic and small bowel cohorts. Overall, scores ≥50 were significantly associated (*P* = 0.0013) with residual disease irrespective of site. The majority (>95%) of patients with SD exhibited scores <65. Scores ≥65 were associated with recurrence, progression, and poorer OSs.

Kaplan-Meier survival curves confirmed the value of this cutoff. The mPFS was 46 (16–87) months in those with NETest2.0 scores ≥65 (Supplemental Fig. S5A, see https://links.lww.com/AOSO/A652). This was associated with χ^2^ = 10.0 (*P* < 0.0001) and an HR of 0.026 (0.011–0.063). The mOS was 99 (57–99) months in those with NETest2.0 scores ≥65 (Supplemental Fig. S5B, see https://links.lww.com/AOSO/A652). This was associated with χ^2^ = 14.88 (*P* < 0.0001) and an HR of 0.16 (0.06-0.40).

## DISCUSSION

This study evaluates the utility of NETest2.0 in surgical management and follow-up of PanNET and SBNET. The assay works effectively for tumors derived from both organ types and in different settings: detection of residual disease (cutoff ≥ 50), prediction of progressive disease (cutoff ≥ 65), or prognostic (OS, cutoff ≥ 65). No significant differences were identified in AUCs between PanNET and SBNET. Multivariable analyses identified that NETEst2.0 appears to be the dominant determinant of modeled outcomes (RFS, PFS, and OS), highlighting its potential utility as a primary biomarker in these settings and supporting further investigation into its role in integrated diagnostic and prognostic frameworks. Different cutoff analyses identified ≥50 for recurrence and ≥65 as a clinically useful boundary for progression. NETest2.0 provides prognostic information independent of conventional clinical variables, while liver metastases remain an important established predictor of OS. The combined significance of these variables suggests that integrating molecular and clinical markers may improve risk stratification in patients with NETs. The study allows us to make some generalizations about patient follow-up and management. NETest2.0 provides a molecular tool with the potential to aid in the management of GEPNET. These findings support a biomarker-guided management strategy, whereby scores could contribute to the reduction of frequency and the number of imaging studies. The assay could replace less accurate tumor markers and allow for early identification of disease recurrence or progression. Changes in scores, particularly increases, were associated with these phenomena. We consider that this liquid biopsy could be used in treatment selection (treat if high score) and for guiding decisions for adjuvant therapy, as is currently suggested in colorectal and pancreatic cancers.^39,40^

In the surgical setting, the tool effectively differentiated between “cures” (R0) versus residual disease who subsequently developed image-detectable disease. In PanNET, >90% of those “cured” or with NED exhibited scores <50. Of note, the majority of these patients did not develop RD. This is consistent with earlier NETest studies, albeit with a shorter follow-up (1–2 years) that identified no recurrence if scores were “normal” post-surgery.^18,35^ The value of the current study is that follow-up was available for up to 12 years. For SBNET, 75% exhibited a negative postsurgical score. None developed recurrence. The 3 who were positive post-surgery all had scores <65. Overall, the ≥50 cutoff exhibited a 100% sensitivity with ≈90% specificity and accuracy for identifying “cures.” It is of interest that postsurgical scores ≥65 were consistently (100%) associated with disease recurrence and lower survival.

This study identified that a patient with an NETest2.0 score ≥65 will almost invariably (≈95% of cases) develop progression irrespective of the tumor origin (pancreas or small bowel). The assay cutoff had good metrics, including a sensitivity of >80% and a specificity of 96%. The latter identifies that the assay may have value as a rule-out for progression. We anticipate that individuals with scores <65 may require less intense and fewer follow-ups, including imaging. The time-to-progression was shorter in PanNET (mPFS = 16 months) than for SBNET (mPFS = 56 months), which likely reflects the differences in natural history of these diseases and/or the follow-up protocol utilized. Of note, higher NETest2.0 scores were typically associated with more aggressive disease (G2, stage IV) although this was more readily evident in PanNET than in SBNET. Grading did not appear to be associated with NETest2.0 scores for small bowel disease (which may reflect the small number of G2), but differences were identified in the pancreatic setting.

In the current study, high NETest2.0 scores (≥65) were associated with a poorer prognosis (mOS, 89–99 months/7–8 years). The metrics were reasonable, with a sensitivity of 67% and a specificity/accuracy of ≈80%. This information is important for both the clinician and patient as it could have utility as a monitor of prognosis. One goal would be to shift the score lower; patients with scores <65 had a >85% chance of being alive after 12 years of follow-up.

One limitation of the study is the relatively small number of patients (n = 29 PanNET; n = 53 SBNET) and the relatively few repeat follow-up samples. Score changes identified surgical efficacy^18,35^ and were postulated to have value for monitoring watch-and-wait or responses to therapy, for example, peptide receptor radionuclide therapy.^41^ Strengths of the study include the >10-year follow-up and the single study site. NETest2.0 scores (and dynamic changes) could be included with clinical features associated with response and outcome. Several authors have developed or suggested such approaches.^42–44^ This study identifies that NETest2.0 scores may be a predominant factor in any such algorithm. CgA measurements were available in 82% of cases in which NETest was performed, with the majority (71%) falling within the normal range, thereby limiting discriminatory utility.

This study evaluates the clinical value of NETest2.0 as a marker for the management of PanNET and SBNET. Scores identified surgical “cures”; individuals were negative. These patients could potentially be followed up less frequently as could patients with disease on imaging or scores <65. Elevated scores (≥65) were associated with disease progression irrespective of management and had long-term prognostic significance. These patients, particularly if they are demonstrating increasing scores and present with clinical evidence of disease progression, should be offered treatment. We consider that NETest2.0 could have value in identifying patient follow-up in the surgical setting and may impact decision-making regarding intervention and frequency of imaging. For example, the combination of imaging and NETest2.0 score could be used as a predictor of outcome (increases in tumor burden/score ≥ 65), or the combination of a normal NETest2.0 score (<50) and negative imaging could be used during the follow-up schedule to minimize the need for imaging.

This biomarker-guided approach reflects established practices in other malignancies, including colorectal cancer, melanoma, and lung cancer. In colorectal cancer, ctDNA-based monitoring predicts recurrence and detects minimal residual disease prior to imaging.^45,46^ In melanoma, ctDNA levels correlate with tumor burden and have been used to inform real-time disease status.^47^ In localized lung cancer, ctDNA profiling allows early detection of minimal residual disease (before radiologic relapse^48^), while, in advanced disease, ctDNA-guided therapy provides early, targeted treatment adjustments that have survival benefits.^49^ Based on these findings, NETest2.0 could enable a clinically actionable management framework.

1. Patients with scores <50 may be considered for reduced surveillance intensity.
2. Patients with scores of 50 to 64 may continue standard monitoring.
3. Patients with scores ≥65 represent a high-risk group for progression and may benefit from intensified imaging or earlier therapeutic intervention.

This risk-adapted approach provides a basis for biomarker-guided clinical decision-making in NET management.

In conclusion, these findings support NETest2.0 as a molecular surveillance assay capable of identifying patients with PanNET and SBNET in durable remission, those with stable disease, and those at imminent risk of progression. Such a strategy may improve progression-free survival by facilitating earlier treatment modification and may reduce healthcare utilization by avoiding unnecessary imaging in low-risk patients. This approach aligns with molecular testing in other cancers and is supported by clinical societies (eg, American Society for Clinical Oncology) that have identified the importance of using circulating biomarkers to guide decision-making and reduce unnecessary interventions while maintaining patient safety.^50^

## Acknowledgments

Anna Malczewska and Gule Hanid were involved in blood sample collection.

## Supplementary Material


