# Supplementary material for: Clinical Value of a 51-Neuroendocrine Tumor–Specific Gene Signature (NETest 2.0) for Management of Pancreatic and Small Bowel Neuroendocrine Tumors: A Prospective Study in a Consecutive Cohort of 82 Patients Considered for Surgery
Source: Ann Surg Open. 2026 Aug 10;7(3):e700. doi: 10.1097/AS9.0000000000000700 (PMC13596935; doi:10.1097/AS9.0000000000000700)
Supplement: Supplementary file 1 [file as9-7-e700-s001.pdf]

**SUPPLEMENTARY METHODS AND RESULTS:**

# Clinical value of a 51 neuroendocrine tumour-specific gene signature (NETest2.0) for management of pancreatic and small bowel neuroendocrine tumors – a prospective study in a consecutive cohort of 82 patients considered for surgery

Frilling A[1*], Clift A[1,2], Spalding D[1], Drymousis P[3], von Roon A[4], Goldin R[1], Bomanji J[5], Wasan H[1], Halim AB[6], Kidd M[6*]

[1]Department of Surgery and Cancer, Imperial College London, London, United Kingdom.
[2]Cancer Research UK Oxford Centre, University of Oxford, Oxford, United Kingdom.
[3]Department of Surgery, Ealing Hospital, London North West University Healthcare NHS Trust, London, United Kingdom.
[4]Department of Surgery, University College London Hospitals NHS Foundation Trust London, London, United Kingdom.
[5]Department of Nuclear Medicine, University College London Hospitals NHS Foundation Trust London, United Kingdom.
[6]Wren Laboratories, Branford, CT, USA

***Corresponding authors**
Mark Kidd, mkidd@wrenlaboratories.com
Andrea Frilling, a.frilling@imperial.ac.uk

## Supplementary Methods

*STARD approach*: The study followed Standards for Reporting of Diagnostic Accuracy Studies (STARD) guidelines [9]. A total of 123 subjects with NET were assessed, of whom 82 (67%) met the inclusion criteria (PanNET or SBNET and baseline sample) and underwent follow-up including image-guided assessments. The STARD diagram illustrating patient selection is included in **Supplementary Figure S1**.

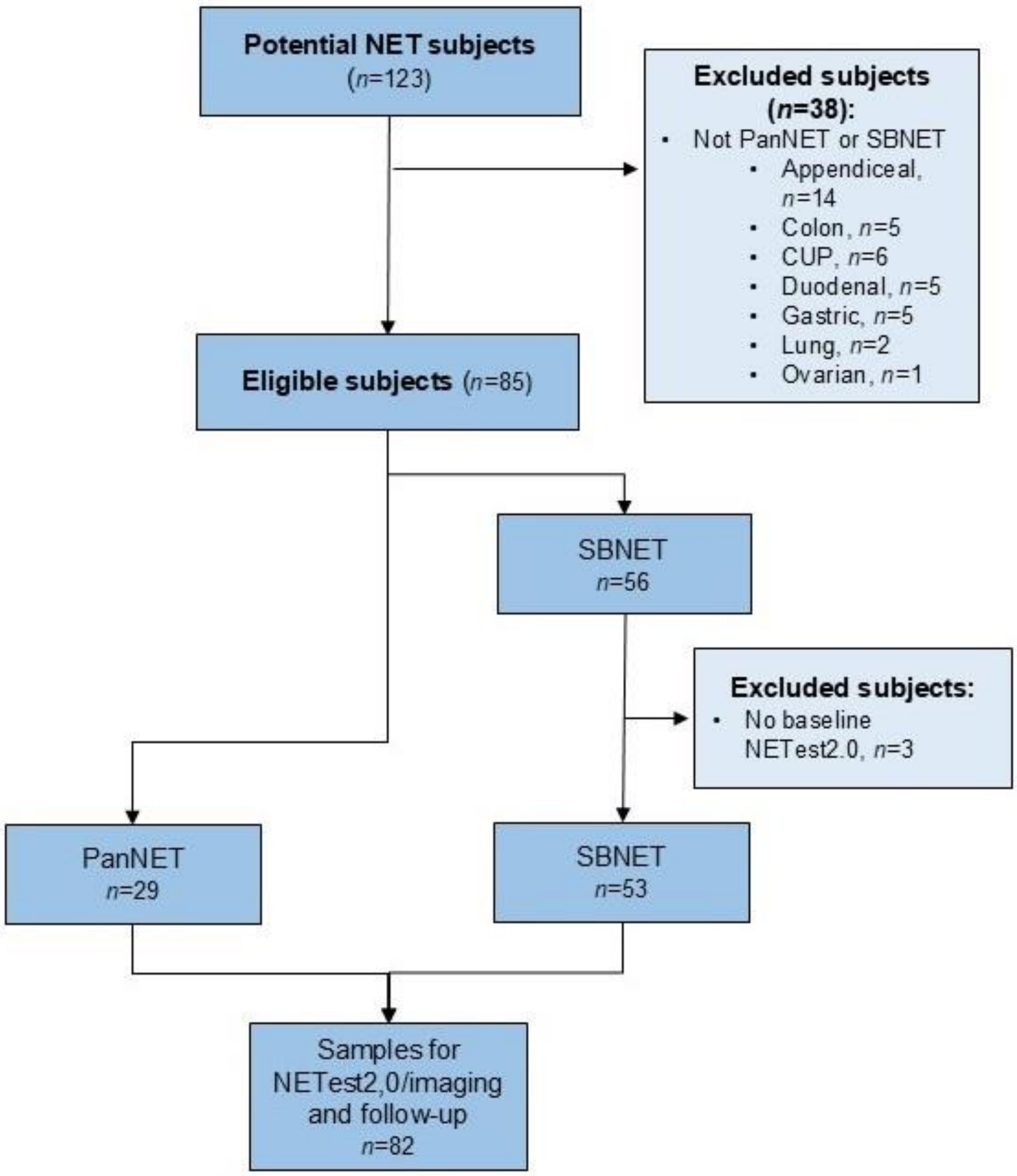


**Supplementary Figure 1**. Overview of the study and outcomes (STARD diagram). No patients were lost-to follow-up.

*Diagnostics:* At baseline and during follow-up, a panel of diagnostic procedures including clinical assessment, imaging (e.g. computed tomography [CT], magnetic resonance imaging [MRI], $^{68}$Gallium($^{68}$Ga)- DOTA-D-Phe1-Tyr3-Thr8-octreotide (DOTATATE) positron emission tomography (PET)/CT, and $^{18}$Fluorine-2-deoxyglucose ($^{18}$F-FDG) PET/CT), endoscopic procedures, and biochemical tests were performed according to standard protocols as described previously [1].

*Chromogranin A:* Although CgA was collected, an audit identified that samples were non-informative and were therefore excluded from analysis. Overall, CgA was measured in 82% of samples (where a NETest2.0 was measured). Of these, 71% were normal which confounded any analytic approach. In addition, in the PanNET cohort, PPI usage confounded values in the 17% that were positive. In the small bowel cohort, only 30% of samples were positive for CgA. Dialysis was a confounder in some instances.

*NETest2.0*: All patients had a baseline NETest2.0 score. Follow-up samples (1 to 4) were available in 61%, 27%, 10% and 5% respectively. We focused on baseline sample and the first follow-up. In illustrative cases, we provide information regarding other NETest2.0 scores if they were available.

*Staging and Grading:* Tumour staging and grading were according to the European Neuroendocrine Tumour Society (ENETS) tumour-node-metastasis (TNM) staging and grading system [2, 3]. The World Health Organisation (WHO) classification 4$^{th}$ Edition was considered [4].

*Treatment:* Surgical and non-surgical treatment was according to ENETS guidelines and under consideration of individual patients' condition. In surgical patients, resection status was determined according to standard R0/R1/R2 criteria.

*Monitoring of Response to Treatment and Follow-up:* Regular physical examination, blood tests and morphologic imaging every 3-6 months, and functional imaging every 6-12 months or anytime earlier if suspicious findings on morphologic imaging were evident. Responses were evaluated with European Organisation for Research and Therapy of Cancer (EORTC) criteria (PET component of PET/CT) as well as by Response Evaluation Criteria in Solid Tumours (RECIST) (CT component of PET/CT or MRI) [5]. Patients were followed up in our clinic until death or study end date (01 September 2025). For overall survival (OS) analyses, all deaths

were censored as “1”, and survivors as “0”. All deaths were related to disease. Follow-up encompassed standard biochemistry, assessment of blood and urinary standard tumour markers for NET, morphologic imaging every 3-6 months, and PET/CT based imaging every 6-12 months.

*Statistical analyses:*

Data are presented as positive/negative, median scores (range) and mean±SD of the 0-100 NETest2.0 score. AUROC analyses were performed using NETest2.0 scores as a continuous variable (scale 0-100) for recurrence free survival (RFS), progression free survival (PFS) and overall survival (OS). Kaplan-Meier curves were developed to evaluate RFS based on a cut-off of ≥50, and curves were developed for PFS and for OS based on a cut-off of ≥65. The 95% confidence intervals are included for survival curves. $Chi^2$ analyses were performed for categorical data. Diagnostics including sensitivity, specificity and accuracy were undertaken. Multivariable analyses (linear regression) and Cox-proportional analyses were undertaken for RDS, PFS and OS. All analyses were performed using Prism version 9.4 for Windows (GraphPad Software Inc, La Jolla, CA, USA, www.graphpad.com) and MedCalc Statistical Software v23.2.1 (MedCalc Software Ltd, Ostend, Belgium; http://www.medcalc.org; 2017. Statistical significance was defined as p<0.05.

**Supplementary Results**

We undertook AUROC analyses for NETest2.0 and RDS, PFS and OS. We evaluated different cut-offs (50-80) to determine which score may have the most value for predicting outcomes. **Supplementary Figure S2** includes the AUCs for RFS (**S2A**), PFS (**S2B**) and OS (**S2C**). These were 0.94, 0.97 and 0.79, respectively.

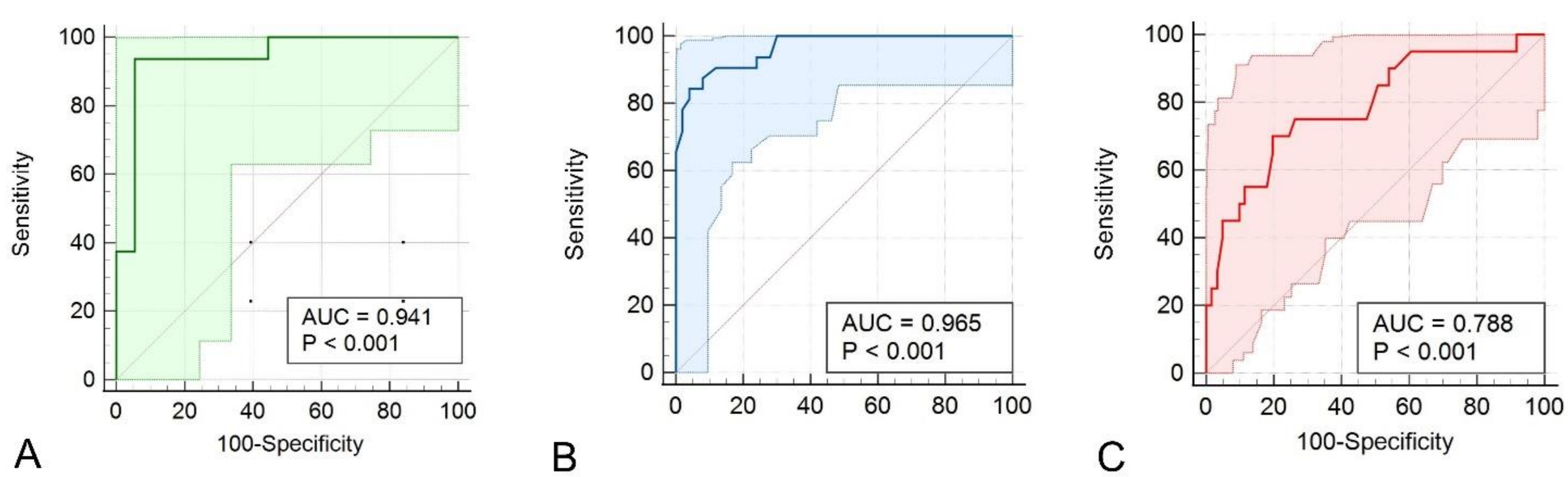


**Supplementary Figure S2**. AUC for NETest2.0 and RFS (**S2A**), PFS (**S2B**) and for OS (**S2C**). 95% CI (shading) is included.

RFS = recurrence free survival, PFS = progression free survival and OS = overall survival.

Diagnostic metrics (sensitivity, specificity and positive likelihood ratio [PLR] extracted from each of the three AUCs are included in **Supplementary Table S1**.

**Supplementary Table S1**. Diagnostic metrics for PFS and OS from NETest2.0 AUCs.

| | ≥50 | ≥55 | ≥60 | ≥65 | ≥70 | ≥75 | ≥80 |
|---|---|---|---|---|---|---|---|
| | **RFS** | | | | | | |
| Sensitivity | 94 (70-100) | 75 (48-93) | 62.5 (35.4-85) | 53 (30-75) | 45.6 (24.7-70) | 36.1 (15-58.7) | 25 (7.3-52.4) |
| Specificity | 89 (65-99) | 94 (73-100) | 94 (73-100) | 94 (73-100) | 94 (73-100) | 100 (82-100) | 100 (82-100) |
| PLR | 8.44 | 13.5 | 11.25 | 9.56 | 8.43 | N/A | N/A |
| | | | | | | | |
| | **PFS** | | | | | | |
| Sensitivity | 100 (89-100) | 90.6 (75-98) | 84.4 (67.2-94.7) | 75.0 (56.6-88.5) | 65.6 (46.8-81.4) | 46.9 (29.1-65.3) | 21.9 (9.3-40) |
| Specificity | 56 (41.3-70) | 82 (68.6-91) | 94 (83.5-98.7) | 98 (89.4-99.9) | 100 (92.9-100) | 100 (92.9-100) | 100 (92.9-100) |
| PLR | 2.27 | 5.03 | 14.06 | 37.5 | N/A | N/A | N/A |
| | | | | | | | |
| | **OS** | | | | | | |
| Sensitivity | 95 (75.1-99.9) | 80 (56.3-94.3) | 75 (80.9-91.3) | 70 (45.7-88.1) | 55.0 (31.5-76.9) | 50.0 (27.2-72.8) | 25.0 (8.7-49.1) |
| Specificity | 26.2 (15.8-39.1) | 50.8 (37.7-63.9) | 68.9 (55.7-80.1) | 80.3 (68.2-89.4) | 82.0 (70-90.6) | 90.2 (79.8-96.3) | 96.7 (88.7-99.6) |
| PLR | 1.29 | 1.63 | 2.41 | 3.56 | 3.05 | 5.08 | 7.62 |

*PLR = positive likelihood ratio*

Survival analysis of the RFS cohort is included in **Supplementary Figure S3**. The median RFS was 8 months (5-17) in those with NETest2.0 scores ≥50. This was associated with a $Chi^2$ = 10.0 (*p*=0.0016) and a hazard ratio of 0.189 (0.067-0.530).

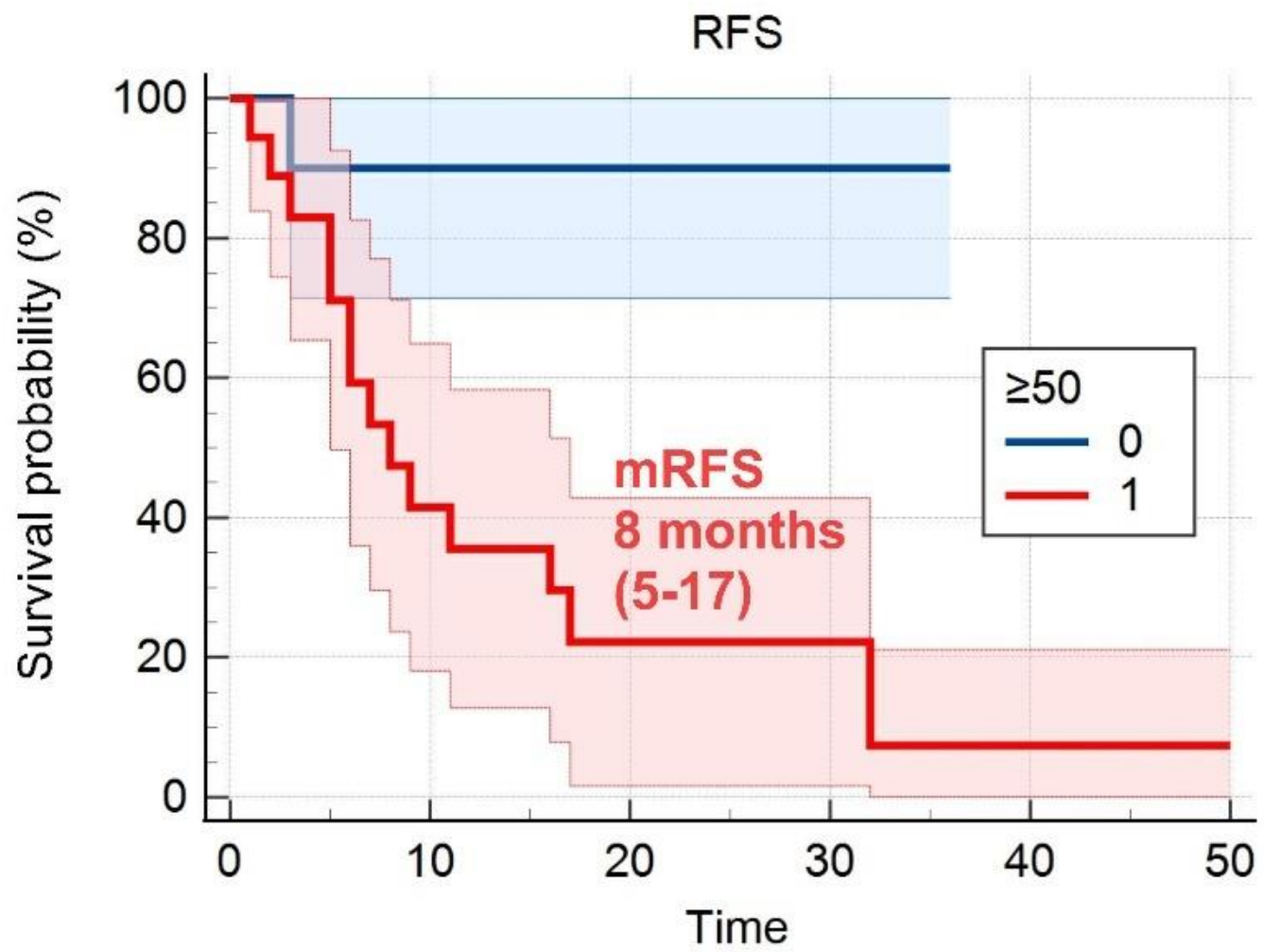


**Comparison of survival curves (Logrank test)**

| | |
|---|---|
| Chi-squared | 10.0021 |
| DF | 1 |
| Significance | P = 0.0016 |

**Hazard ratios with 95% Confidence Interval**

| Factor | No recurrence | Recur |
|---|---|---|
| ≥50 | - | 5.3082<br>1.8865 to 14.9357 |
| <50 | 0.1884<br>0.06695 to 0.5301 | |

**Supplementary Figure 3.** RFS survival curve in PanNET and SBNET (*n*=33).
Kaplan-Meier survival scores identifying a cut-off of ≥50 significantly differentiated those with no evidence of disease (NED: blue) versus those that recurred (red). Log-rang Chi2 and hazard ratios are included.

We next undertook both a multivariable linear regression analysis as well as multivariable Cox-regression analysis with multiple inputs (grade/stage/score/residual disease) to evaluate the impact of the NETest2.0 as well as other factors on outcomes (RFS, PFS and OS).

## Multivariable analysis

**Supplementary Table S2** includes the outputs from linear regression analyses. In multivariable linear regression analysis, NETest2.0 score emerged as the only independent predictor of the RFS. The F-ratio was 7.914 (*p*=0.0001) and Shapiro Wilk test: 0.977 (*p*=0.691).

**Supplementary Table S2**. Multivariable regression analysis (RFS)

| Independent variables | Coefficient | Std. Error | 95% CI | T | P | $r_{partial}$ | $r_{semipartial}$ |
|---|---|---|---|---|---|---|---|
| **(Constant)** | -0.9418 | 0.3855 | -1.7315 to -0.1520 | -2.4427 | **0.0211** | | |
| **Tumor type (PanNET vs. SBNET)** | 0.2855 | 0.1542 | -0.03029 to 0.6013 | 1.8519 | 0.0746 | 0.3303 | 0.2253 |
| **Grade** | -0.9418 | 0.3855 | -1.7315 to -0.1520 | -2.4427 | **0.0211** | -0.9418 | 0.3855 |
| **Liver metastases** | 0.02503 | 0.1562 | -0.2949 to 0.3450 | 0.1603 | 0.8738 | 0.03027 | 0.0195 |
| **LN metastases** | 0.0907 | 0.1819 | -0.2819 to 0.4633 | 0.4987 | 0.6219 | 0.09383 | 0.06067 |
| **NETest2.0 score** | 0.02081 | 0.004294 | 0.01202 to 0.02961 | 4.8465 | **<0.0001** | 0.6754 | 0.5896 |

**Supplementary Table S3** includes information from Cox proportional modelling. NETest2.0 score was independently associated with increased hazard (HR 1.07 per unit increase, 95% CI 1.026–1.12, $p$=0.0018). Tumor type was also significant ($p$=0.0136) but was highly variable. No other clinical variables, were significantly associated with RFS.

**Supplementary Table S3**. Multivariable Cox regression analysis (RFS)

| Covariate | β | SE | Wald | P | Exp(b) | 95% CI of Exp(b) |
|---|---|---|---|---|---|---|
| **Tumor type (PanNET vs. SBNET)** | 1.666 | 0.675 | 6.0915 | **0.0136** | 5.291 | 1.4091 to 19.8665 |
| **Grade** | 1.1785 | 0.7387 | 2.5454 | 0.1106 | 3.2495 | 0.7639 to 13.8222 |
| **LM** | -0.2387 | 0.6229 | 0.1469 | 0.7016 | 0.7876 | 0.2323 to 2.6703 |
| **LN** | -0.6583 | 0.8517 | 0.5973 | 0.4396 | 0.5177 | 0.0975 to 2.7487 |
| **NETest2.0 score** | 0.06987 | 0.02236 | 9.7607 | **0.0018** | 1.0724 | 1.0264 to 1.1204 |

**Supplementary Table S4** includes the outputs from linear regression analyses. In multivariable linear regression analysis, NETest2.0 score emerged as the only independent predictor of the PFS. The F-ratio was 22.404 ($p$<0.0001) and Shapiro Wilk test: 0.984 ($p$=0.452).

**Supplementary Table S4**. Multivariable regression analysis (PFS)

| Independent variables | Coefficient | Std. Error | 95% CI | T | P | $r_{partial}$ | $r_{semipartial}$ |
|---|---|---|---|---|---|---|---|
| **(Constant)** | -0.6625 | 0.3225 | -1.3050 to -0.01998 | -2.0545 | **0.0435** | | |
| **Tumor type (PanNET vs. SBNET)** | -0.03258 | 0.08806 | -0.2080 to 0.1429 | -0.3699 | 0.7125 | -0.04296 | 0.02435 |
| **G1** | -0.1597 | 0.2358 | -0.6296 to 0.3102 | -0.6773 | 0.5003 | -0.07849 | 0.04458 |
| **G2** | 0.1045 | 0.2281 | -0.3499 to 0.5589 | 0.4582 | 0.6482 | 0.05318 | 0.03016 |
| **LM** | 0.09523 | 0.084 | -0.07214 to 0.2626 | 1.1337 | 0.2606 | 0.1307 | 0.07462 |
| **LN** | 0.1043 | 0.1022 | -0.09931 to 0.3080 | 1.0208 | 0.3107 | 0.1178 | 0.06719 |
| **Residual disease** | -0.06773 | 0.1011 | -0.2691 to 0.1337 | -0.6701 | 0.5049 | -0.07766 | 0.0441 |
| **NETest2.0 score** | 0.01928 | 0.002657 | 0.01399 to 0.02457 | 7.2566 | **<0.0001** | 0.6448 | 0.4776 |

**Supplementary Table S5** includes information from Cox proportional modelling. NETest2.0 score was independently associated with increased hazard (HR 1.08 per unit increase, 95% CI 1.047–1.114, p < 0.0001). No other clinical variables, including tumor grade, primary site, lymph node involvement, or liver metastases, were significantly associated with PFS.

**Supplementary Table S5**. Multivariable Cox regression analysis (PFS)

| Covariate | β | SE | Wald | P | Exp(b) | 95% CI of Exp(b) |
|---|---|---|---|---|---|---|
| **Tumor type (PanNET vs. SBNET)** | 0.5866 | 0.464 | 1.5979 | 0.2062 | 1.7978 | 0.7240 to 4.4640 |
| **G1** | -1.0981 | 0.8813 | 1.5523 | 0.2128 | 0.3335 | 0.0593 to 1.8764 |
| **G2** | -0.5744 | 0.8463 | 0.4606 | 0.4973 | 0.5631 | 0.1072 to 2.9575 |
| **LM** | 0.3873 | 0.4236 | 0.836 | 0.3605 | 1.473 | 0.6422 to 3.3787 |
| **LN** | - 0.06057 | 0.818 | 0.005483 | 0.941 | 0.9412 | 0.1894 to 4.6776 |
| **Residual disease** | 13.6258 | 256.6687 | 0.002818 | 0.9577 | 827234.1 | 2.7342E-213 to 250.2786E+222 |
| **NETest2.0 score** | 0.07697 | 0.01577 | 23.8133 | **<0.0001** | 1.08 | 1.0471 to 1.1139 |

For OS, both NETest2.0 score and liver metastases (LM) were identified as independent predictors of overall survival (**Supplementary Table S6**). The F-ratio was 5.106 ($p$=0.0001) and Shapiro Wilk test: 0.962 ($p$=0.017). NETest2.0 scores demonstrated a statistically significant positive association with the outcome ($p$=0.005) as did liver metastases ($p$=0.0135). In contrast, other clinicopathologic variables—including primary tumor type (PanNET vs. SBNET), tumor grade (G1, G2), lymph node involvement, and residual disease—were not significantly associated with overall survival (all $p > 0.05$). Although G1 demonstrated a trend toward significance ($p$=0.093), its confidence interval crossed zero, indicating insufficient evidence for an independent effect.

**Supplementary Table S6**. Multivariable regression analysis (OS)

| Independent variables | Coefficient | Std. Error | 95% CI | t | P | $r_{partial}$ | $r_{semipartial}$ |
|---|---|---|---|---|---|---|---|
| **(Constant)** | -0.9552 | 0.3925 | -1.7375 to -0.1730 | -2.4337 | 0.0174 | | |
| **Tumor type (PanNET vs. SBNET)** | 0.01621 | 0.1153 | -0.2136 to 0.2460 | 0.1406 | 0.8886 | 0.01646 | 0.01348 |
| **G1** | 0.4969 | 0.2922 | -0.08539 to 1.0792 | 1.7007 | 0.0932 | 0.1952 | 0.1631 |
| **G2** | 0.4214 | 0.2881 | -0.1528 to 0.9955 | 1.4626 | 0.1479 | 0.1687 | 0.1403 |
| **LM** | 0.2789 | 0.1102 | 0.05929 to 0.4986 | 2.531 | **0.0135** | 0.284 | 0.2427 |
| **LN** | 0.0024 | 0.1312 | -0.2592 to 0.2640 | 0.01829 | 0.9855 | 0.002141 | 0.001754 |
| **Residual disease** | 0.06385 | 0.1209 | -0.1771 to 0.3048 | 0.5280 | 0.5991 | 0.06168 | 0.05063 |
| **NETest2.0 score** | 0.009389 | 0.003247 | 0.002917 to 0.0158 | 2.8914 | **0.005** | 0.3206 | 0.2773 |

**Supplementary Table 7** includes information from Cox proportional modelling. NETest2.0 score and liver metastases (LM) were identified as independent predictors of overall survival. NETest2.0 score was significantly associated with an increased hazard of death (HR = 1.056, 95% CI: 1.016–1.098, p = 0.0057), indicating that each unit increase in NETest2.0 score corresponds to an approximately 5.6% increase in mortality risk. The presence of liver metastases was also significantly associated with worse survival (HR = 4.11, 95% CI: 1.08–15.60, p = 0.0376), consistent with its established role as a major adverse prognostic factor. Patients with liver metastases had more than a fourfold increased risk of death compared to those without. No other clinical variables, including tumor grade, primary site, or lymph node involvement were significantly associated with PFS.

**Supplementary Table S7**. Multivariable Cox regression analysis (OS)

| Covariate | β | SE | Wald | P | Exp(b) | 95% CI of Exp(b) |
|---|---|---|---|---|---|---|
| **Tumor type (PanNET vs. SBNET)** | 0.2921 | 0.5727 | 0.2601 | 0.6101 | 1.3392 | 0.4358 to 4.1151 |
| **G1** | 15.2548 | 701.2273 | 0.000473 | 0.9826 | 4217703 | 0.0000 to 10.1423E+303 |
| **G2** | 14.5464 | 701.2272 | 0.00043 | 0.9834 | 2076898 | 0.0000 to 10.1423E+303 |
| **LM** | 1.4141 | 0.6801 | 4.3237 | **0.0376** | 4.1126 | 1.0845 to 15.5952 |
| **LN** | -0.1829 | 0.8723 | 0.04398 | 0.8339 | 0.8328 | 0.1507 to 4.6035 |
| **Residual disease** | 12.7276 | 212.3887 | 0.003591 | 0.9522 | 336933.4 | 5.4785E-176 to 2.0722E+186 |
| **NETest2.0 score** | 0.0549 | 0.01986 | 7.6398 | **0.0057** | 1.0564 | 1.0161 to 1.0984 |

Low scores (<50) reliably identified patients without residual or recurrent disease (**Supplementary Figure 4**). These patients could undergo routine follow-up with reduced imaging. Post-surgical patients with no evidence of disease (ED) but raised scores (≥50-65) could be considered for adjuvant therapy. Patients with ED and scores of ≥50–65 should be considered for standard surveillance. Scores ≥65 predicted progression with high specificity. Under these circumstances, early/earlier imaging could be considered with treatment reassessment (if progression is demonstrated at imaging). This enables risk-adapted follow-up, including potential reduction of imaging intensity in molecularly stable patients and earlier therapeutic intervention in high-risk individuals.

**Recurrence**

| NETest2.0 Score | RD | NED |
|---|---|---|
| <50 | 0 | 20 |
| 50-64 | 3 | 3 |
| ≥65 | 7 | 0 |

Chi$^2$=10.38, ***p*=0.0013**
Sensitivity: **100%**
Specificity: **87%**
Accuracy: **91%**

**Progression**

| NETest2.0 Score | PD | SD |
|---|---|---|
| <50 | 0 | 8 |
| 50-64 | 5 | 18 |
| ≥65 | 17 | 1 |

Chi$^2$=27.66, ***p*<0.0001**
Sensitivity: **81%**
Specificity: **96%**
Accuracy: **89%**

**Overall Survival**

| NETest2.0 Score | Dead | Alive |
|---|---|---|
| <50 | 1 | 15 |
| 50-64 | 6 | 34 |
| ≥65 | 14 | 11 |

Chi$^2$=16.82, ***p*<0.0001**
Sensitivity: **67%**
Specificity: **82%**
Accuracy: **78%**

**Supplementary Figure 4.** Summation of scores (different cut-offs) and outcomes in both cohorts (*n*=82). *We focused on recurrence, progression and overall survival. Chi$^2$ and diagnostic metrics are included. For recurrence, we used the cut-off of normal (<50); for progression and overall survival, we used a cut-off of ≥65.*

Survival analysis of the PFS and OS cohorts are included in **Supplementary Figure S5**. The median PFS was 46 months (16-87) in those with NETest2.0 scores ≥65 (**S5A**). This was associated with a Chi$^2$ = 10.0 ($p$<0.0001) and a hazard ratio of 0.026 (0.011-0.063). The median OS was 99 months (57-99) in those with NETest2.0 scores ≥65 (**S5B**). This was associated with a Chi$^2$ = 14.88 ($p$<0.0001) and a hazard ratio of 0.16 (0.06-0.40).

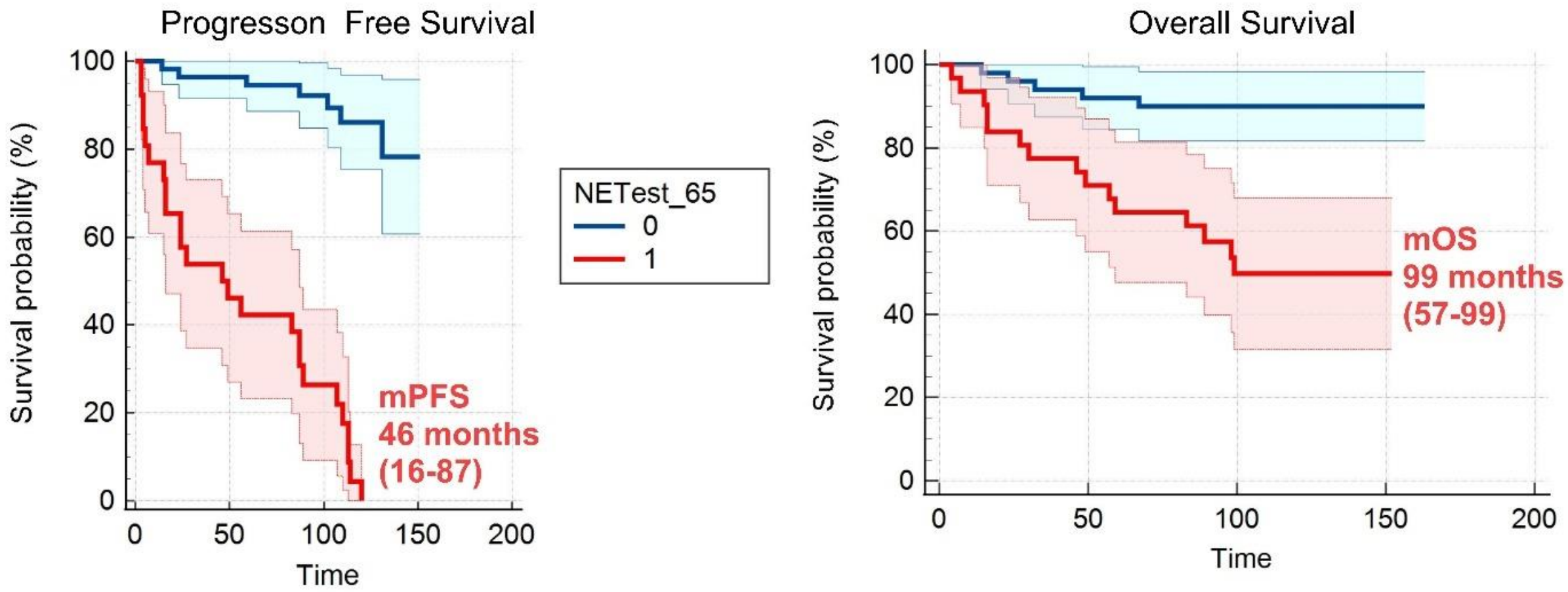


| | |
|---|---|
| Chi-squared | 66.6957 |
| DF | 1 |
| Significance | P < 0.0001 |

| Factor | <65 | ≥65 |
|---|---|---|
| Progress | - | 37.8125 15.8127 to 90.4199 |
| Stable | 0.02645 0.01106 to 0.06324 | - |

A

| | |
|---|---|
| Chi-squared | 14.8822 |
| DF | 1 |
| Significance | P = 0.0001 |

| Factor | <65 | ≥65 |
|---|---|---|
| Dead | - | 6.1853 2.4507 to 15.6107 |
| Alive | 0.1617 0.06406 to 0.4080 | - |

B

**Supplementary Figure 5**. Survival curve for PFS (**S5A**) and OS (**S5B**) (*n*=82).
Kaplan-Meier survival scores identifying a cut-off of ≥65 significantly differentiated progression (red) from stable disease (blue) and those that survived (blue) from those who died (red). Log-rank $Chi^2$ and hazard ratios are included.

## References


1. Clift AK, Kidd M, Bodei L, et al. Neuroendocrine Neoplasms of the Small Bowel and Pancreas. *Neuroendocrinology* 2020; 110(6):444-476. doi: 10.1159/000503721. Epub 2019 Sep 27.
2. Rindi G, Kloppel G, Alhman H, et al. TNM staging of foregut (neuro)endocrine tumors: a consensus proposal including a grading system. *Virchows Arch.* 2006; 449(4):395-401. Epub 2006 Sep 12.
3. Rindi G, Kloppel G, Couvelard A, et al. TNM staging of midgut and hindgut (neuro) endocrine tumors: a consensus proposal including a grading system. *Virchows Arch.* 2007; 451(4):757-62. Epub 2007 Aug 3.
4. Lloyd RV, Osamura RY, Kloppel G, et al. WHO classification of tumours of endocrine organs. 4 ed. Vol. 10: WHO Press, 2017.
5. Eisenhauer EA, Therasse P, Bogaerts J, et al. New response evaluation criteria in solid tumours: revised RECIST guideline (version 1.1). *Eur J Cancer* 2009; 45(2):228-47.
